# Blocking Dopaminergic Signaling Soon after Learning Impairs Memory Consolidation in Guinea Pigs

**DOI:** 10.1371/journal.pone.0135578

**Published:** 2015-08-14

**Authors:** Kiera-Nicole Lee, Sanika Chirwa

**Affiliations:** 1 Department of Neuroscience and Pharmacology, Meharry Medical College, 1005 DB Todd Boulevard, Nashville, TN, 37208, United States of America; 2 Department of Pharmacology, 23^rd^ Avenue South & Pierce, Vanderbilt University, Nashville, TN, 37203, United States of America; University of Toronto, CANADA

## Abstract

Formation of episodic memories (i.e. remembered experiences) requires a process called consolidation which involves communication between the neocortex and hippocampus. However, the neuromodulatory mechanisms underlying this neocortico-hippocampal communication are poorly understood. Here, we examined the involvement of dopamine D1 receptors (D1R) and D2 receptors (D2R) mediated signaling on memory consolidation using the Novel Object Recognition (NOR) test. We conducted the tests in male Hartley guinea pigs and cognitive behaviors were assessed in customized Phenotyper home cages utilizing Ethovision XT software from Noldus enabled for the 3-point detection system (nose, center of the body, and rear). We found that acute intraperitoneal injections of either 0.25 mg/kg SCH23390 to block D1Rs or 1.0 mg/kg sulpiride to block D2Rs soon after acquisition (which involved familiarization to two similar objects) attenuated subsequent discrimination for novel objects when tested after 5-hours in the NOR test. By contrast guinea pigs treated with saline showed robust discrimination for novel objects indicating normal operational processes undergirding memory consolidation. The data suggests that involvement of dopaminergic signaling is a key post-acquisition factor in modulating memory consolidation in guinea pigs.

## Introduction

In humans memory is defined as the ability to acquire, store, and retrieve information. Several different types of long-term memory (LTM) have been distinguished, including declarative and non-declarative memory. Declarative memory is further subdivided into episodic memory (i.e. experiences and events) and semantic memory (i.e. facts, meanings, and concepts) which both require conscious recall. Thus episodic memory is the term given to the capacity to recall or ‘remember’ experienced events and situations [1]. Once thought to be unique to humans, it is now clear that the core behavioral properties of episodic memory are present across mammals as well as other animal species [2]; the major brain regions responsible for episodic memory in humans have anatomical and functional homologs in other species [2, 3]. Significant progress has been made in our understanding of the operational features of the neural circuits underlying memory but there is still much that is unknown about the establishment of LTM in the brain. For example, encoding of newly acquired information into engrams (i.e. memory traces) is fast and may occur on a single trial. However, memories are initially labile and later become resistant to loss but the processes that make short-term memories (STM) take on a permanent form are not well understood.

The formation of episodic memory critically depends on the integrity of the hippocampus but also involves a large network of cortical areas that includes the adjacent parahippocampal region and the prefrontal cortex [2, 4–5]. It is theorized and supported by experimental data that ‘consolidation’ is a process by which initially labile memories become permanent and impervious to disruption [6, 7]. Memory consolidation is commonly addressed at two complementary levels of description and analysis namely the cellular/synaptic level (synaptic consolidation) and the brain systems level (systems consolidation) [6, 8]. Consolidation is separated into a molecular-cellular process of ‘fixation’ of a memory trace that occurs for several minutes after learning [9–11], and a time-dependent ‘reorganization’ of neural networks resulting in episodic memory storage [7, 12–18]. Thus, current research is being directed towards the discovery of specific patterns of neuromodulatory activity underpinning system memory consolidation [19]. Here, we used guinea pigs as an animal model to verify or refute the neuromodulatory role of dopaminergic signaling on memory consolidation.

We sought to check the involvement of dopaminergic signaling in memory consolidation for several reasons. Accumulating evidence implicates endogenous dopamine from dopaminergic neurons in the ventral tegmental area (VTA) as a key regulator of synaptic changes observed at certain stages of learning and memory and of synaptic plasticity in CA1 area of the hippocampus [20, 21]. Specifically, the VTA and hippocampus are theorized to form a functional loop designed to detect novelty. This novelty signal would then serve as a gate to convert behaviorally relevant STM into LTM [21]. Consistent with this postulate are findings showing that novel stimuli trigger burst firing of VTA cells [22–24] which send projections to the hippocampus [24, 25]. This dopaminergic novelty signal from the VTA is presumably detected by D1/D5 receptors that are expressed in hippocampal pyramidal cells [26–28]. In the CA1 region, D1/D5 receptors have previously been reported to modify electrically induced CA3/CA1 long-term potentiation (LTP: a cellular correlate of mnesic process [29]) and LTM formation when drug antagonists were administered prior to learning [30–34]. However, studies have yet to distinguish whether the impact of dopaminergic signaling is limited to the learning (acquisition) phase or if it also affects processes after learning, i.e. consolidation. We present in this report data showing that blocking dopaminergic receptors after learning impairs novel object recognition (NOR) memory in guinea pigs.

## Materials and Methods

### Animals and Drug Administration

Forty-two male Hartley guinea pigs (outbred; weight 200–250 g) obtained from Charles River Laboratories were housed in pairs without environmental enrichment (i.e. no toys or objects); they had free access to food and water. Guinea pigs were kept on a 12-hour light/dark cycle (lights on at 7:00 AM) in the American Association for Laboratory Accreditation Council certified Animal Care Facility (ACF) at Meharry Medical College. The ACF maintains animal rooms at 65–70°F and 40–70% relative humidity. After adjustment to the ACF for 7 days, guinea pigs housed in pairs were later assigned to 3 experimental groups (n = 14 per group) for treatment with the following: (a) phosphate buffered saline (PBS: hereafter denoted as saline), the control group, (b) SCH23390, the dopamine D1 receptor (D1R) antagonist [35, 36], or (c) sulpiride, the dopamine D2 receptor (D2R) antagonist [36]. To make drug solutions, 10 mg SCH23390 or 20 mg sulpiride were first dissolved in 0.5 mL dimethyl sulfoxide (DMSO) and then 0.5 mL saline was added to make final solution volumes of 1.0 mL respectively. These stock solutions were further diluted with saline to provide drug concentrations of either 0.25 mg/kg/mL SCH23390 or 1.0 mg/kg/mL sulpiride that were the doses administered to guinea pigs, respectively. Thus, the dilution factor for DMSO in the final 1.0 mL injections given to animals ranged between 1:20 and 1:40 which corresponds to 0.01225 μM–0.025 μM DMSO (molecular weight of DMSO = 78.13). The DMSO concentrations we used are below those that have been utilized by other investigators but did not affect novel object recognition (NOR) performance in control animals. For example, Rossato and co-workers tested local DMSO concentrations that were ~12.8 mM (i.e. 0.1% DMSO in saline; molecular weight of DMSO = 78.3) and this apparently did not modify NOR performances in their controls animals [37]. NOR tests the animals’ natural instinct to explore new objects more than familiar objects, and can, therefore, test its recognition memory [38]. In our hands, it is unlikely the systemic DMSO concentrations exposed to guinea pigs ever attain DMSO concentrations as cited above in view of the further dilution caused by the large blood volume of guinea pigs, i.e. 20 mL for a guinea pig weighing approximately 300 g [39]. Sulpiride, DMSO and phosphate buffered tablets were obtained from Sigma-Aldrich (St. Louis, MO), whereas SCH23390 was acquired from Tocris (Ellisville, MO).

### Guinea Pig Activity Assessments

**Fig 1:**
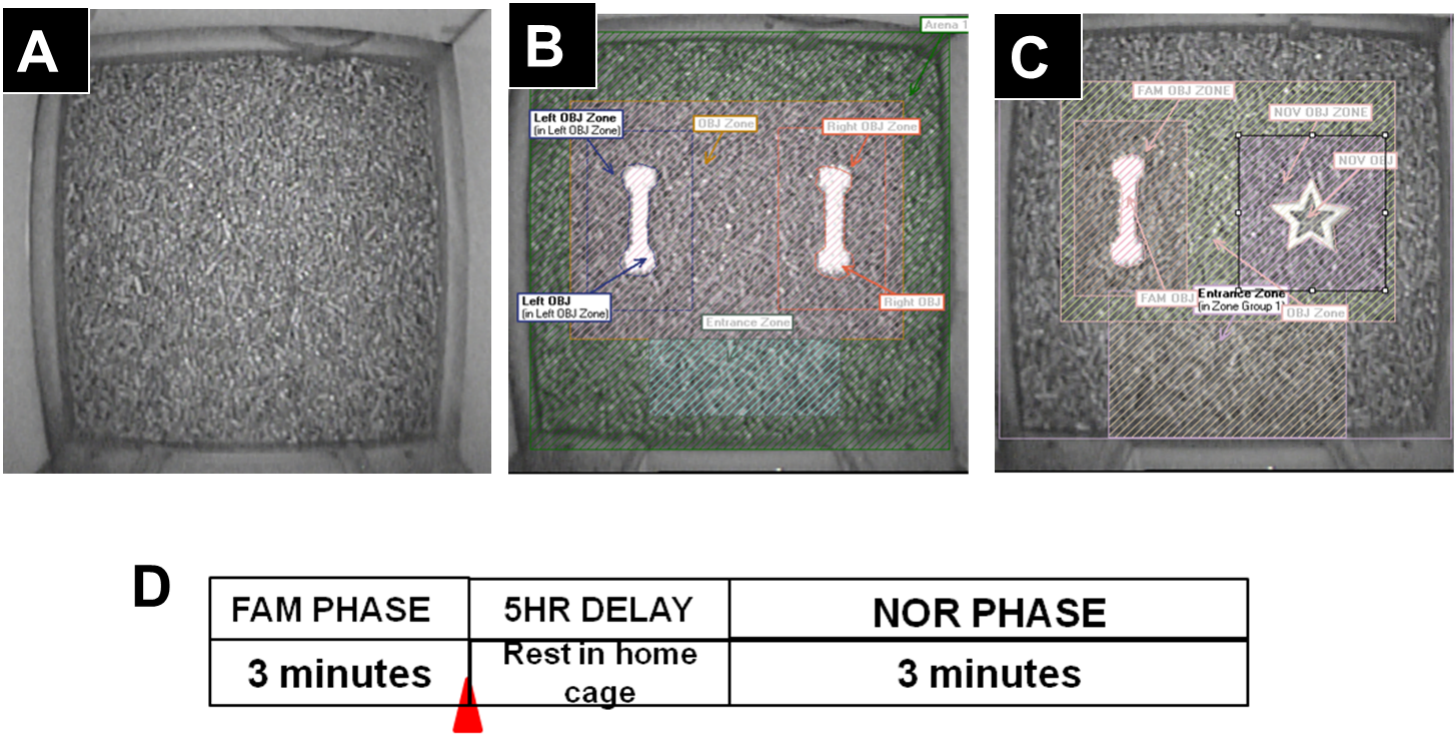
Phenotyper cages from Noldus used to conduct novel object recognition tests. In (A) is shown the top view of the Phenotyper cage configured as an open arena for animal habituation prior to the start of the object recognition testing as described in the main text. This was proceeded with the addition of two familiar objects (B) followed by replacement of one familiar object and one novel object (C). Note that the objects were placed centrally in all cases. Furthermore, the different zones in the cage were digitally demarcated using EthoVision XT 7 acquisition and analyses software. Automated recordings could be conducted continuously unobtrusively and with experimenter out of sight. In (D) is shown the experimental protocol that was followed to conduct the NOR tests as described in detail in the text.

When performing behavior experiments, it is important for the testing times to match the animal’s arousal state and this was done in the present study. Behavior experiments were conducted in the Phenotyper multi-purpose observation cage from Noldus that is constructed of four transparent polymethylmethacrilate (PMMA) walls and a PMMA dual satin opaque cage bottom (cage dimensions in cm: 45 long x 45 wide, 65 high). The cage bottom was covered with Cell-Sorb Plus bedding (Fig 1) made from recycled paper and is odor free (Mednet Direct Farmington, CT). The Phenotyper cage had a top unit that contained an infrared (IR) sensitive camera and operated with EthoVision XT 7.0 video-tape and data acquisition and analysis software. The EthoVision XT 7.0 programs were used to electronically demarcate user defined boundaries of the open arena and the objects within the arena (Fig 1B and 1C). This video-tracking software automatically tracked guinea pigs activities and this data was used to quantify time spent exploring objects and distance moved while undertaking the NOR tests as described below.

### Novel Object Recognition Test

After adjustment in the ACF for 7 days, guinea pigs underwent habituation for 5 days in the empty Phenotyper cage (Fig 1A). Hence, guinea pigs explored an open arena with floor bedding in the Phenotyper cages for 3 min, as described previously by Clark and Squire [40] with some modifications. Subsequently, on Day-6, guinea pigs were brought back to the experiment room between 8:00 AM–9:00 AM and each guinea pig was placed in the empty Phenotyper cage with floor bedding for 1 min to re-habituate to the cage. Guinea pigs were removed and two of the same objects (denoted as FAM1 and FAM2) were placed in the center regions of the cages, about 4–7 cm away from the cage side walls and 2–3 cm apart (Fig 1B). Following a ~2 min interval, guinea pigs were brought back to the Phenotyper cages and allowed to spontaneously explore two identical objects for 3 min. This was the familiarization phase (denoted as FAM) and represented the learning phase for the two identical objects. Immediately after the FAM phase, guinea pigs were randomly assigned to different treatment groups and given acute intraperitoneal injections of one of the following: a) 1 ml phosphate buffered saline, denoted as ‘saline’ from hereon, n = 14; b) 0.25 mg/kg SCH23390, n = 14; or c) 1 mg/kg sulpiride, n = 14. It should be noted that the basic method of administering dopaminergic antagonists immediately after training to study consolidation of object recognition memory has been used by other investigators [37]. In our study, guinea pigs were processed in batches of 6–10 animals. In each case, a pair of animals was randomly assigned to the saline group whereas the remainder were assigned to SCH23390 and sulpiride groups. To avoid possible time-dependent effects when drugs were administered, we ensured that all treatments were conducted at the same time-points during the day. After the injections, guinea pigs were returned to their home cages (with free access to food and water) and taken back to the ACF for an interval of 5 hours before novel object recognition tests were done. During the 5-hour interval, one familiar object (FAM1 or FAM2; Fig 1B) was randomly exchanged with a novel object (denoted as NOV; Fig 1C). After the 5-hour interval, guinea pigs returned to the same Phenotyper cages for the test phase, to explore the two objects (i.e. familiar and novel) for 3 min. Guinea pig activities during the five days of habituation, familiarization, and novel object recognition testing were video-tracked using the EthoVision XT 7.0 software. The experimenter stayed in the behavior lab during these recordings but was out of sight to the guinea pigs. Between experiments the Phenotyper cages (including objects) were cleaned with soap and hot water and disinfected with 70% isopropyl alcohol and the floor bedding was replaced in between animals to prevent cross contamination.

We quantified total distances moved by each guinea pig during the first three minutes of both the familiarization and testing phases to detect any differences in locomotor activity among the treatment groups. Then, we determined time spent exploring each object (i.e. FAM1, FAM2 and NOV) for the first 3 minutes during the different phases as applicable. Last, we calculated the discrimination index (DI) particularly between NOV and FAM as follows:

$$\text{DI}=\frac{\text{Time exploring NOV}\;–\;\text{Time exploring FAM}}{\text{Total Time exploring NOV}\;+\;\text{FAM}}$$

### Euthanasia

At the end of experiments, guinea pigs were euthanized with an overdose of pentobarbital (150 mg/kg; source IP: Henry Schein).

### Ethics Statement

All guinea pigs were utilized at Meharry Medical College in accordance with American Veterinary Medical Association (AVMA) Guidelines for the Euthanasia of Animals and approved by the Institutional Animal Care and Use Committee (Protocol Number: 060619JGT10201).

### Statistical Analysis

The data was analyzed using GraphPad Prism 5 for Windows, GraphPad Software, San Diego, California, USA, www.graphpad.com. One-way ANOVA with Tukeys post-hoc test or two-way ANOVA (Model I) with Bonferroni’s post-hoc test were used to compare variables of interest. Comparisons between two groups were done with paired t-test. Data fitness for Gaussian distribution was verified with D’Agostino and Pearson Omnibus normality test [41]. We evaluated 42 guinea pigs for the NOR tests, and at α = 0.05, this had a statistical power equal to 80% sufficient to detect a 7.52 sec difference in object preference with a standard deviation of ± 2.63 sec.

## Results

### Guinea Pigs Exhibit Thigmotaxic Behavior

**Fig 2:**
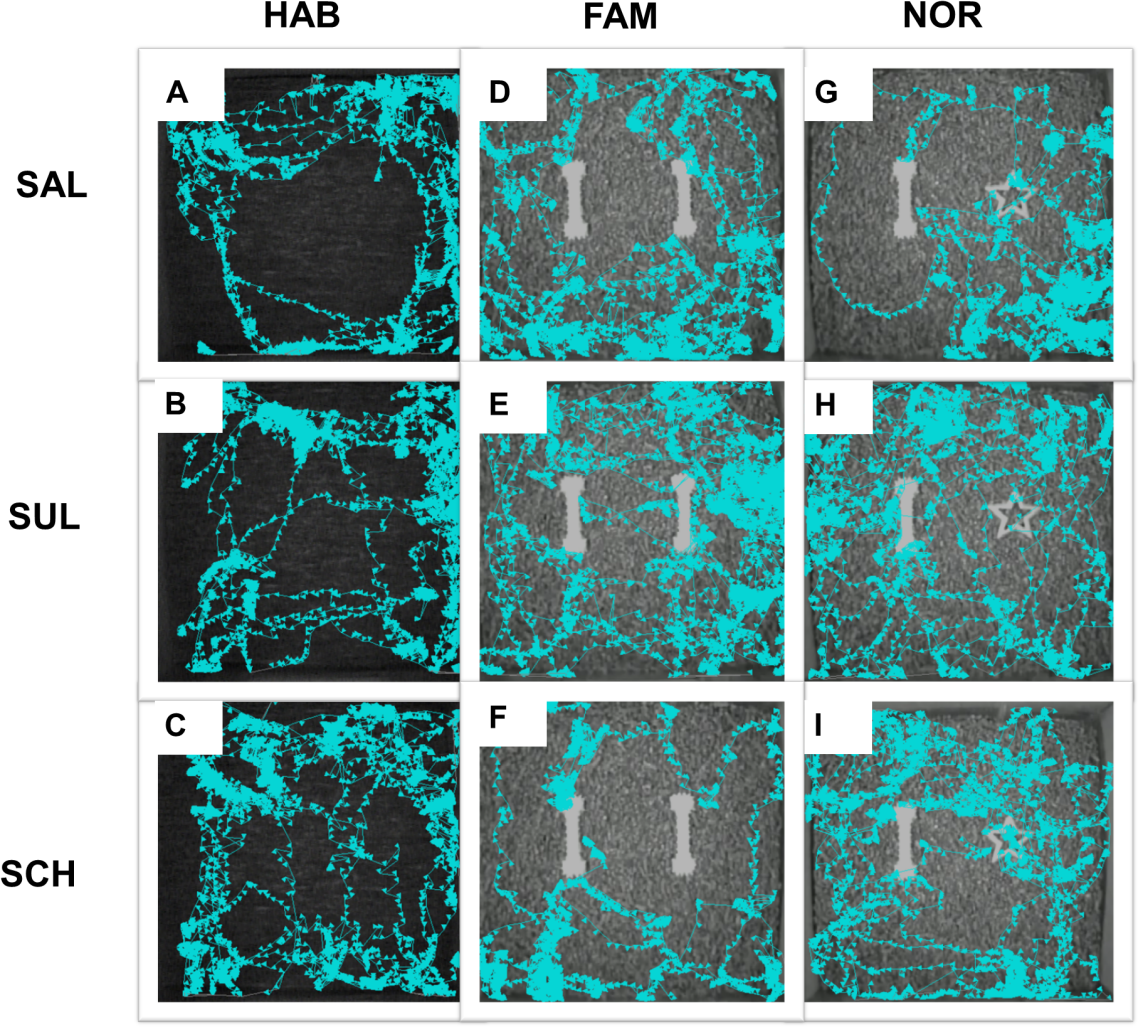
Activity profiles recorded from representative guinea pigs from the treatment groups. Each row presents data from the same guinea pig from the indicated treatment group whereas columns reflect the different behavior phases assessed. In each case, EthoVision XT 7.0 software tracked the guinea pig activities using a three point detection system (i.e. nose, center of body, and rear) but only the nose tracks (light blue with arrow head denoting head direction) are shown in the records. In (A-C) note the heavy ‘traffic’ in peripheral regions relative to the central portions of the cage during habituation. During the familiarization phase (D-F) guinea pigs explored two similar objects equally well and this was prior to drug administrations. In (G-F) are shown track marks following drug treatments–i.e. soon after familiarization phase–and then a 5-hour interval. Note that the guinea pig treated with saline spent more time with the novel object (G), whereas the guinea pig treated with sulpiride (H) or SCH23390 (I) did not show preferences for the novel object (star-shaped object) relative to the familiar object (bell-bar object). Abbreviations: SAL, saline group; SUL, sulpiride group, and SCH, SCH23390 group.

The current study investigated the involvement of dopaminergic signaling on memory consolidation after learning using the NOR test in the guinea pig animal model. A recent study from the laboratory showed that guinea pigs remained active throughout daytime and nighttime but showed peak activities at dusk–i.e. lights off set at 7:00 PM [42]. Consequently, the behavioral experiments in the current study were started in the morning (8:00 AM–9:00 AM) each day such that the test phase for NOR occurred in the afternoon between 1:00 PM–3:00 PM respectively. The results showed that the average distance travelled by guinea pigs was 762.8 ± 38.8 cm during the habituation phase (values are mean ± SEM in this and subsequent entries). Data fitness for Gaussian distribution was confirmed using the D’Agostino and Pearson omnibus normality test (K2 = 1.362, p = 0.5061, N = 42 guinea pigs). Guinea pigs typically stayed close to the walls (i.e. displayed thigmotaxis behaviors) rather than the center regions in the Phenotyper cages. Therefore, objects were placed in the central sectors of the cages, which were less frequently visited, to best capture 'purposeful' exploration of objects in the NOR experiments (Fig 2).

### Blocking Dopamine D1Rs or D2Rs Signaling with Antagonists Impairs Recognition Memory Formation

**Fig 3:**
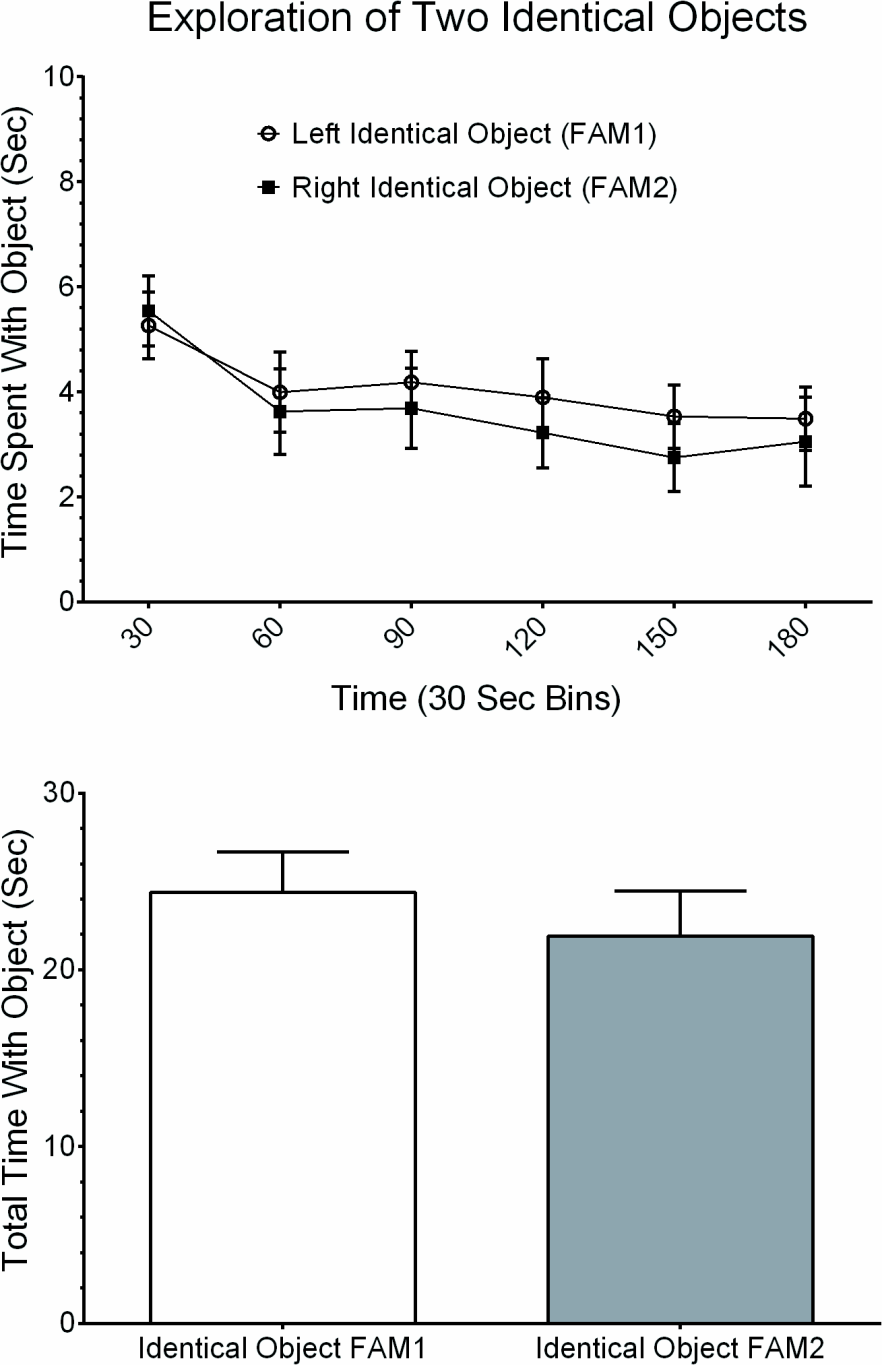
Time spent with two similar objects during familiarization phase. In (A) is group data showing the amount of time guinea pigs spent with the left (unfilled circles) and right (filled squires) objects of the same type. Data is given in 30 second bins and the recording was for 3 minutes. Each plotted point is mean ± SEM. In (B) is summary of group data showing there were insignificant differences between the total time spent exploring the left and right objects during the 3 minute observation period (paired t(41) = 0.8845, p > 0.05).

After concluding the 5-day habituation phase, experiments started with guinea pigs being re-habituated in the Phenotyper cages for 1 minute. Animals were then removed to allow placements of two identical objects in the cages (i.e. FAM1 and FAM2, Fig 1B) and returned back into the cage and video-tracked for 3 minutes. Guinea pigs explored the two identical objects equally well during the familiarization phase and this was characterized by animals touching and sniffing the objects (Fig 2). Plots of time spent exploring the left (FAM1) and the right (FAM2) identical objects, assessed in 30-sec time intervals, are shown in Fig 3A. The two-factor analysis of variance showed no significant differences in time spent with either object, F(1, 41) = 0.7819, p = 0.3817; no significant differences for the interaction between time interval and time spent with either object, F(5, 205) = 0.2359, p = 0.9463; but the time interval factor was significant, F(5, 205) = 2.770, p = 0.0191. Furthermore, guinea pigs spent the highest time exploring the two objects within the initial 30 seconds time interval but this tapered down with progression of time in the cage (Fig 3A). Altogether, the results indicated guinea pigs spent the same amount of total time exploring FAM1 (24.37 ± 2.29 sec) as they did exploring FAM2 (21.89 ± 2.58 sec), (paired t(41) = 0.8845, p = 0.3816; Fig 3B).

**Fig 4:**
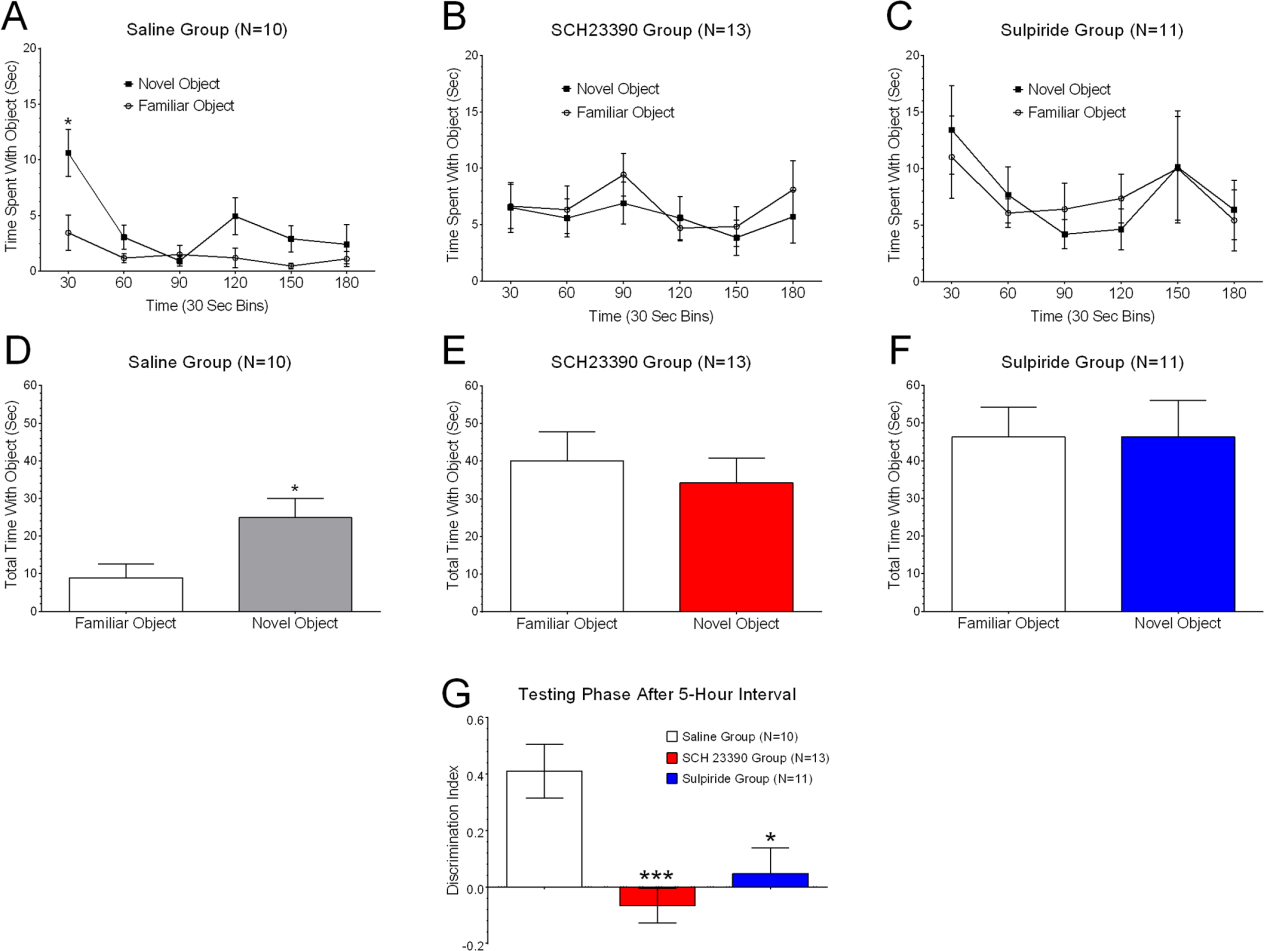
Blocking D1R and D2R with selective antagonists impairs object discrimination. Guinea pigs from the saline group (A) spent a significantly greater proportion of time exploring novel objects within the initial 30 seconds time interval but this tapered down over the 3-minute test window. By contrast, guinea pigs from both the SCH23390 group (B), or the sulpiride group (C), did not exhibit this characteristic feature during the initial 30 second time interval or any other interval over the 3-minute observation period. Note that the object exploration time does not evolve with time in the SCH23390 animal group which reflects the failure of habituation response during the test. Only guinea pigs in the saline group spent significantly more cumulative time with the novel object versus the familiar object (D), whereas there were insignificant differences in time spent between the novel and familiar objects in the SCH23390 group (E) or the sulpiride group (F). Group data (G) for calculated discrimination index revealed significant preferences for novel objects relative to familiar objects in guinea pigs injected with saline, but not SCH23390 or sulpiride [F(2, 32) = 8.791; p = 0.0008; within subject 1-way ANOVA with Tukeys post-hoc tests]. Only guinea pigs that exhibited the same preferences for FAM1 and FAM2 objects during the familiarization phase were used here. Asterisks indicate statistical differences and further discussed in text.

Immediately after the familiarization phase was over, guinea pigs were randomly assigned to separate treatment groups and administered one of the following intraperitoneal injections: a) 1 ml saline, n = 14; b) 0.25 mg/kg SCH23390, n = 14, or c) 1 mg/kg sulpiride, n = 14. After a 5-hour interval each guinea pig was brought back to the Phenotyper cage and underwent testing for novel object recognition memory. The results obtained from the NOR tests for guinea pigs in the three treatment groups are shown in Fig 4. It was important that we evaluated only those guinea pigs that had exhibited similar preferences for FAM1 and FAM2 during the familiarization phase. Consequently, z-scores were computed for time spent with FAM1 and FAM2 and used to detect ‘outliers’ which were guinea pigs with z-scores that exceeded three standard deviations from the mean. Thus, 35 out of the 42 guinea pigs met the criterion for inclusion in final data analyses (10 saline, 13 SCH23390, and 12 sulpiride) and these were evaluated for recognition memory at the 5-hour test interval.

Starting with the saline group, the two-factor analysis of variance showed significant differences in the following factors: a) time spent with the novel object versus the familiar object, F(1, 9) = 8.695, p = 0.0022; b) time interval, F(5, 45) = 9.711, p < 0.0001; and c) the interaction between time interval and time spent with objects, F(5, 45) = 2.707, p = 0.032. As was the case during the familiarization phase, guinea pigs spent a greater proportion of time exploring objects within the initial 30 seconds time interval but this tapered down over the 3-minute test window in the saline group (Fig 4A). By contrast, guinea pigs in the SCH23390 group had no significant preference for the novel object over the familiar object, F(1, 12) = 1.045, p = 0.3268; no significant effect for the time interval factor, F(5, 60) = 1.143, p = 0.3481; and no significance effect for the interaction between object and time interval, F (5, 60) = 0.3604, p = 0.8735. In fact, guinea pigs in this group did not exhibit the characteristic feature of mostly exploring objects in the initial 30 second time interval as seen with the saline group or during the familiarization phase (Fig 4B). Similar results to those seen with the SCH23390 group were obtained in the sulpiride group where the two-way ANOVA yielded an F ratio of F(1, 10) = 0.0007, p = 0.9790, indicating that the preference for the novel object was not significantly different than for the familiar object (Fig 4C). The main effect of time interval in this group was not significant, F(5, 50) = 1.029; the interaction effect of object and time interval was also not significant, F(5, 50) = 1.045, p = 0.4021. This meant guinea pigs in the sulpiride group explored the two objects (novel and familiar) with similar frequencies throughout the 3-minute testing interval (Fig 4C). Overall, only guinea pigs in the saline group spent significantly more time with the novel object than the familiar object (paired t(9) = 4.247, p = 0.0022; data is in Fig 4D), whereas there were no significant differences in time spent between the novel and familiar objects in the SCH23390 group (paired t(12) = 1.022, p = 0.3269; data in Fig 4E) or the sulpiride group (paired t(10) = 0.0286, p = 0.9778; data is in Fig 4F). Finally, the calculated discrimination index (DI) confirmed that only guinea pigs in the saline group (DI score: 0.4130 ± 0.0947), but not the SCH23390 group (DI score: -0.0657 ± 0.0615) or sulpiride group (DI score: 0.0475 ± 0.0896), had a significant DI confirming the development of object recognition memory in the saline group (F(2, 32) = 8.791, p = 0.0008; one-way ANOVA with Tukey’s post hoc test; Fig 4G). Thus, we concluded blocking dopaminergic receptors soon after learning interfered with the development of object recognition memory.

### Acute Administrations of Dopaminergic Receptor Antagonists Do Not Alter Locomotor Activity

**Fig 5:**
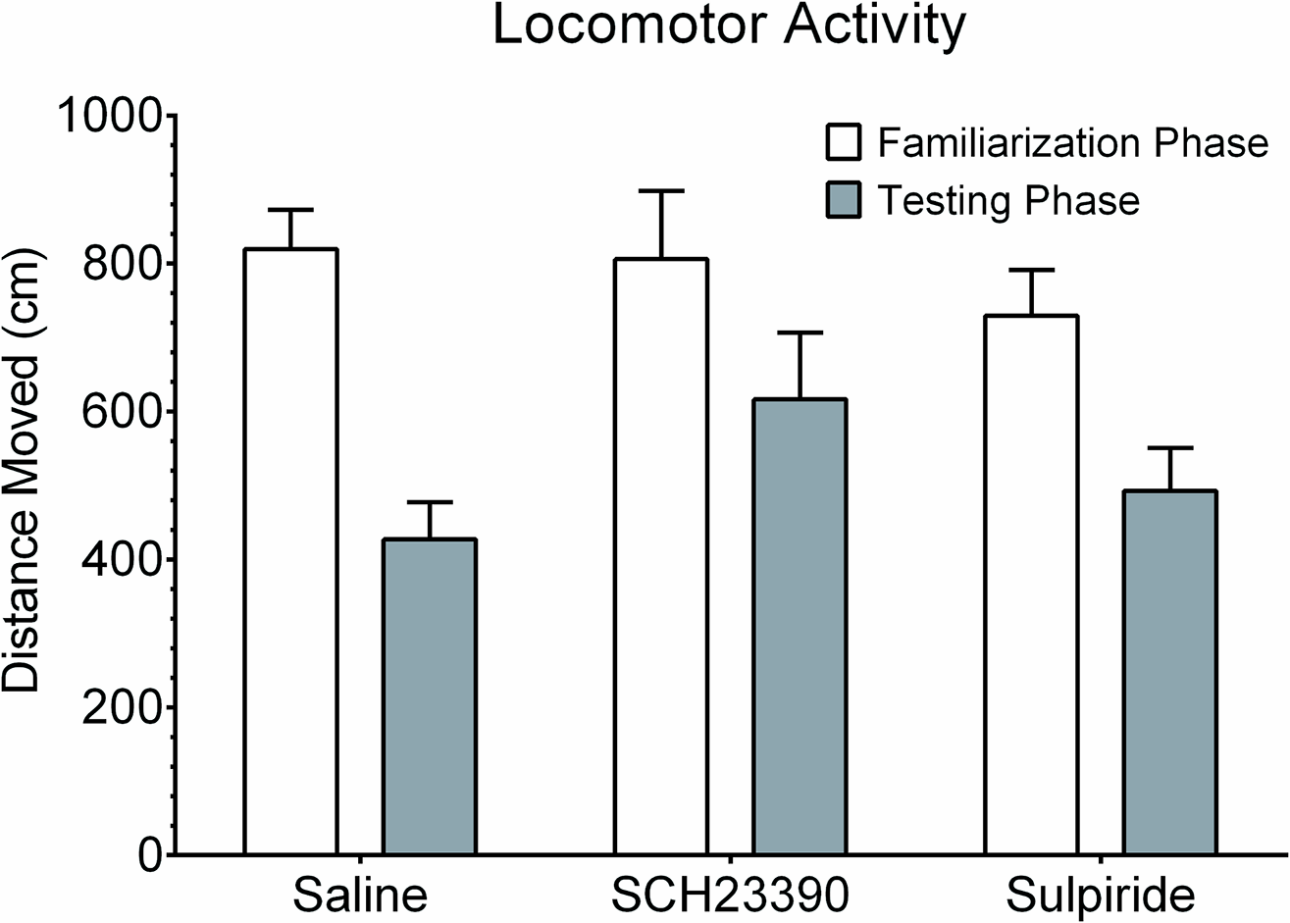
Changes in locomotor activity in the three treatment groups. Data shows that the total distances travelled during the testing phase versus the testing phase was significantly less in the saline group, but not in both the SCH23390 group and sulpiride group. The findings are suggestive of the wearing down of the ‘novelty’ for the new object in the control guinea pigs with progression of time in the observation cage.

One non-cognitive plausible explanation of the above mentioned results is that both SCH23390 and sulpiride could have affected locomotor activity which then curtailed movement during the NOR testing phase. To check this, an assessment of the distances moved during the familiarization (pre-drug) phase and the testing (post-drug) phase of the NOR assay was performed. The data from this comparison is shown in Fig 5. The dependent one way analysis of variance showed significant differences in total locomotor activities between the NOR phases, F(5. 64) = 4.715, p = 0.001. Post hoc analyses using the Tukey’s post hoc criterion for significance indicated that the average locomotor activity during the testing phase was significantly less in the saline group, but was not significantly different in both the SCH23390 group and sulpiride group. In fact the saline group quickly explored the novel and familiar objects in the initial 30 second interval and then decreased perhaps indicating a rapidly developing ‘habituation’ to the two objects. On the other hand, guinea pigs in both the SCH23390 group and the sulpiride group kept exploring the two objects (novel and familiar) equally well for nearly the entire 3 minutes.

## Discussion

We have successfully utilized the guinea pig animal model to assess the involvement of D1R and D2R mediated signaling on object recognition memory consolidation. A key feature of these experiments was that the competitive antagonists used to block D1Rs or D2Rs signaling (i.e. SCH23390 and sulpiride respectively) were administered soon after (but not before) the learning phase. The primary finding is that blocking dopaminergic signaling soon after learning impairs recognition memory consolidation in the guinea pig animal model. We injected drugs via the intraperitoneal route partly to mimic the majority of therapeutic drugs that are administered systemically in humans and, therefore, has translational relevance. For instance, sulpiride administered orally in humans is known to be significantly more effective than haloperidol, risperidone and olanzapine in schizophrenic treatment [43]. However, only limited information is available on the potential risks associated with sulpiride treatment on higher cognitive functions [44]. Certainly, our method for drug administration did not permit us to localize the predominant site(s) in the brain where the antagonists exerted their effects to cause changes in memory consolidation. Nonetheless, our findings set the stage for designing studies that will examine dopaminergic antagonists applied in specific brain targets to better localize the integrative sites of drug actions. The above findings are discussed in detail below.

First, guinea pigs treated with the two dopaminergic antagonists clearly did not exhibit preferences for novel objects relative to familiar objects after a 5-hour test interval. By contrast, saline-treated animals showed significant preferences for novel objects within 30 seconds of entering the cage. This is in agreement with published work that has reported that animals tend to explore novel objects within 30 seconds of being exposed to the object [40]. Thus, we concluded that blocking dopaminergic signaling with drug antagonists soon after learning induced disruptions of processes undergirding memory consolidation. Second, we applied drug doses that we and others have found to block late-LTP [45], alter cAMP levels [46], and/or interfere with memory formation [28] when administered prior to learning. We predict that the drugs undergo rapid distribution after an intraperitoneal injection and reach pharmacologically relevant concentrations in the brain within minutes. We do not know the half-lives of SCH23390 or sulpiride in the guinea pig model but it is probable that their optimal effects span over the 30–60 min interval in view of the following. Our published data, taken together with reports in the literature, indicate that late-LTP (or even memory tests) are attenuated in animals injected with SCH23390 ~30 min prior to tetanic stimulation [45] or learning phase in NOR studies [28]. This suggests a relatively long duration of action after acute injections in vivo. However, the effects of the drug antagonists would be absent or minimal after the 5-hour interval from acute administration when NOR tests were conducted. In a separate study, we found that SCH23390 and sulpiride lacked effects on motor activities as early as 2 hours after acute drug administration [42]. Third, it was intriguing that two dopaminergic antagonists that primarily work on receptors with opposing effects on the regulation of cAMP, PKA, Ca^++^ levels [47], and ultimately the synthesis of proteins, could elicit the same behavioral phenotypes of memory impairment. However, the data is consistent with published reports that have indicated that infusion of sulpiride in the nucleus accumbens impaired memory consolidation [48]. Studies have reported that blocking D2Rs in the nucleus accumbens caused memory impairment during various task requiring recognition, attention, and retrieval [49–52]. By contrast, rats infused with SCH23390 into the lateral ventricles subsequently were found to be slow learners of novel information in hippocampal-dependent memory tasks [28]. Consequently, it is possible sulpiride and SCH23390 exerted their effects in different brain regions that still led to disruption of memory consolidation. Within this context, it was interesting to discover that object exploration times did not evolve with time in the SCH23390 animal group which probably reflected absence of habituation during the NOR test phase. By comparison, guinea pigs in the sulpiride group exhibited signs of habituation during exploration time but this was not different between the familiar versus the novel object. Taken together, we speculate that SCH23390 might have interfered with ‘working memory’ whereas sulpiride altered ‘short-term memory’ but these ideas require experimental validation. Fourth, we found guinea pigs to be robust small animal models with unique features that render them well suited for assessments of mnemonic functions and behaviors. They are neither nocturnal nor diurnal [42, 53] and, therefore, do not require reversal of light–dark cycles often necessary when using mice or rats. Their relatively consistent exploratory activity allows for unambiguous characterization of behaviors. For example, an increasing number of studies in the literature report that animal attentiveness is a prerequisite for the development of robust memory consolidation [54, 55]. This trait is prominent in guinea pigs making them useful animal models for measuring unambiguous cognitive behaviors. Taken together, our study provides support for the post-learning involvement of D1R and D2R signaling in memory consolidation in the guinea pig animal model and this is consistent with recent findings reported in other rodent species [37, 56–57].

In the broader context, our study has provided additional evidence for the involvement of dopamine D1 and D2 receptors signaling in the modulation of memory consolidation. Other neuromodulators that have been implicated in published reports include acetylcholine [58], GABA [59] and norepinephrine [60]. Taken together, this information contributes towards a better understanding of the neurochemical processes underlying memory consolidation leading to stable LTM. New memories are initially represented within the hippocampus and then become integrated into existing memories in the neocortex during consolidation [61] to form updated memory networks termed ‘schema’ [62–64]. Strong experimental evidence suggests that the prefrontal cortex ‘dictates’ schema development and updating [65]. Overall, the neural pathways for mnemonic information flow between the hippocampus and prefrontal cortex are reasonably well understood [64, 66, 67]. What is less known are the interactive nature of the neuromodulators and related transduction cascades underlying the various steps during memory consolidation. Indeed, much is already known about the involvement of locus ceruleus noradrenergic (LC-NA) system in orchestrating memory consolidation after initial learning [68, 69]. The LC-NA system has been shown to be critically involved in the late phase of memory consolidation and engagement of the prelimbic regions of the prefrontal cortex [70–72]. Thus, β-adrenergic receptors have been found to be necessary for reconsolidation after reactivation of well-established memories of varying emotional valences [73–75]. The question is what then might be the nature of interaction between the DA we have reported in our study and NA systems to support consolidation? Sara and co-workers proposed that the LC-NA outputs contribute towards the transition from the ‘down-state’ (i.e. hyperpolarization) to ‘up-state’ (i.e. depolarization) in cortical structures [76]. The facilitating effects of the LC-NA input on cortical excitability [77, 78], spike timing and synaptic plasticity in cortex [76, 79], and hippocampus [80] are well known. It is tempting to infer that the LC-NA spearheads the ‘dialogue’ between brain structures that produce LTM formation. We further speculate that a mechanism is required to link on-going cortical activity and protein synthesis that, in turn, underpins enduring system consolidation in LTM. We have previously presented data indicating that conditioning stimulation in guinea pig hippocampus led to D1R activation that correlated with both LTP maintenance and up-regulation of growth associated protein 43 (GAP-43) transcript and protein expression [45]. It is conceivable that the LC-NA system helps to prime the down-to-up cortical state and that dopaminergic system helps to sustain the cortical up-state as well as couple this activity to protein synthesis essential for LTM formation. This hypothesis should be further explored in future investigations.
